# Pathogenicity and virulence of *Aspergillus fumigatus*

**DOI:** 10.1080/21505594.2023.2172264

**Published:** 2023-02-15

**Authors:** Kayleigh Earle, Clara Valero, Daniel P. Conn, George Vere, Peter C. Cook, Michael J. Bromley, Paul Bowyer, Sara Gago

**Affiliations:** a Manchester Fungal Infection Group, Faculty of Biology, Medicine and Health, The University of Manchester, Manchester, UK; b MRC Centre for Medical Mycology, University of Exeter, Exeter, UK

**Keywords:** Aspergillus fumigatus, Aspergillosis, Virulence factors, Pathogenicity, at-risk factors

## Abstract

Pulmonary infections caused by the mould pathogen *Aspergillus fumigatus* are a major cause of morbidity and mortality globally. Compromised lung defences arising from immunosuppression, chronic respiratory conditions or more recently, concomitant viral or bacterial pulmonary infections are recognised risks factors for the development of pulmonary aspergillosis. In this review, we will summarise our current knowledge of the mechanistic basis of pulmonary aspergillosis with a focus on emerging at-risk populations.

## Introduction

Respiratory infections caused by fungi, bacteria, and viruses rank fourth in the global causes of death according to the World Health Organisation [1]. Yet, despite fungal respiratory infections causing as many deaths as Influenza or Tuberculosis annually, they remain overlooked [2–4]. There are more than 600 fungal species known to cause infections in humans but it is *Aspergillus* spp. which are responsible for 70% of fungal-associated deaths [2]. Of the more than 200 species of *Aspergillus* that have been identified, only a few are considered to be pathogenic to humans [5].

### Aspergillus

*Aspergillus* species are filamentous fungi, which are prevalent in the environment. The majority of *Aspergillus* spp. live as saprophytes and are frequently isolated from soil and decaying vegetation, where they play an important part in the recycling of nutrients such as carbon and nitrogen [5,6].

*A. fumigatus* is characterised by its small (2–3 μm), blue-green echinulate conidia, which extend in long chains from conidiophores reaching from the vegetative mycelium [7,8]. *A. fumigatus* cells are surrounded by a polysaccharide-based cell wall, which provides physical protection and structural support [9]. Primarily composed of α-1,3-glucan, galactofuran, and mannan, the fungal cell wall is a dynamic structure, which changes in response to the external environment [10]. Shepardson et al. [11] demonstrated that in response to stress, e.g. a hypoxic environment, *A. fumigatus* can alter its transcriptome to upregulate the production of β-glucan to thicken its cell wall and provide protection.

**Figure 1.**
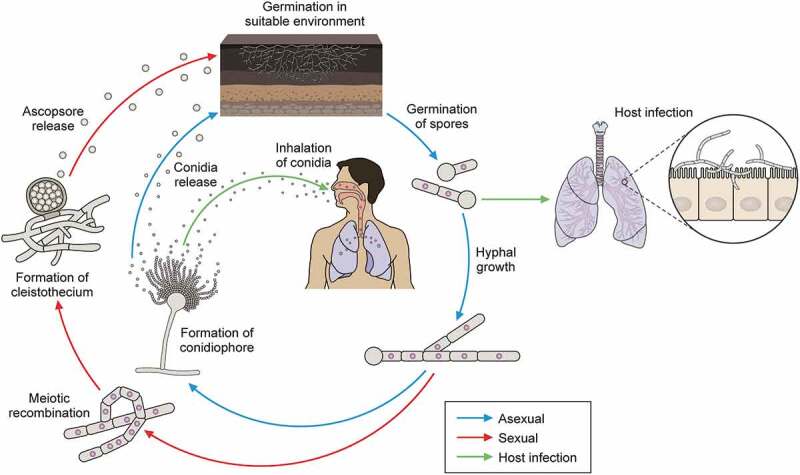
*A. fumigatus* lifecycle both in the environment and a human host. *A. fumigatus* can enter an asexual (blue) or sexual (red) reproductive cycle. During the sexual cycle, the fungus undergoes meiotic recombination to form the cleistothecium, which contains ascospores. Dead plant matter is an ideal environmental niche for *C. fumigatus* to sporulate and grow. During the asexual cycle, the mycelium generates spores, which can be aerially dispersed. Some of these conidia land in a new ecological niche, while others are inhaled by humans and can cause disease (green).

Although it was long believed that *A. fumigatus* was an exclusively asexually reproducing organism, it is now accepted that *A. fumigatus* is also capable of sexual reproduction [12,13]. Nevertheless, the majority of reproduction in nature occurs asexually, with *A. fumigatus* sporulating profusely to generate small, hydrophobic conidia that can be dispersed aerially across significant distances [6] (Figure 1).

Their ubiquity, coupled with their morphology, means that conidia are frequently inhaled by humans and animals [14]. It is estimated that humans inhale 500–5000 fungal spores daily [15–17]. Conidia are extremely tolerant and are capable of withstanding a range of environmental stressors, including temperature, pH, and osmotic pressure [18]. When inhaled by a healthy host, resting conidia are cleared via innate immune defences including mucociliary clearance and phagocytosis before they can cause disease [19,20]. However, if this process is unsuccessful, conidia can undergo a range of morphological changes, driven by periods of isotropic and later polarised growth, to form hyphae which are capable of invading host tissues and causing disease [19,21,22]. Despite germination being heavily implicated in invasive fungal disease, the specific mechanisms behind the transition from resting conidia to hyphae have not yet been fully elucidated [23]. Our current knowledge relies on transcriptomic studies, which have revealed that transcription within conidia can be modulated in response to changes in the environment, altering gene expression when dormancy is broken. Lamarre et al. [24] reported that more than 25% of the total *A. fumigatus* genome is present as transcripts in dormant conidia, with expression of these being regulated to alter fitness, including tolerance of stress and antifungals, as well as virulence and secondary metabolite production [25]. For example, comparative analysis between *Aspergillus sp*. revealed that several conidial stress-related genes were regulated by AtfA [26]. Further work showed that this gene had a role in maintaining conidial dormancy, with atfA deletion mutants undergoing abnormal exits from dormancy and germinating in the absence of nutrients [26].

The risk for the development of pulmonary *Aspergillus*-related disease is significantly elevated in patients with impaired lung defences, which can arise from immunosuppressive treatments such as chemotherapy or from chronic respiratory conditions (e.g. asthma, chronic obstructive lung disease, cystic fibrosis) where the immune system may also be impacted [27–29]. Yet, despite the constant exposure of humans to *A. fumigatus*, only a relatively small subset of the population will develop *Aspergillus*-related illnesses [30].

The severity of *Aspergillus* diseases can range from a mild sensitisation to life-threatening, with rates of prevalence and mortality differing among at-risk populations [2]. Diagnosis of aspergillosis is often delayed due to non-specific symptoms, low sensitivity of microbiological culture and a lack of globally available non-culture-based methods and, this further contributes to poor patient prognosis [29,31–33]. First-line treatment for pulmonary aspergillosis currently relies on the use of azole drugs e.g. voriconazole and itraconazole. Echinocandins like caspofungin and the polyene amphotericin B have also been used as second-line treatments for pulmonary aspergillosis with varying levels of success. However, treatment failure due to host intolerance or the emergence of antifungal resistance or adaptation is a common outcome [34–38].

Our understanding of aspergillosis has changed dramatically in the last decade, with the importance of the host condition and concomitant infections becoming increasingly recognised. Yet, despite a coordinated effort from the scientific community to understand the pathophysiology of pulmonary aspergillosis, it is still unclear why aspergillosis does not occur universally in at-risk populations. Similarly, we do not understand how *A. fumigatus* adapts within the host to cause different clinical presentations of the disease or to overcome the pressure of antifungal activity.

In this review, we will focus on *A. fumigatus* pathogenicity, highlighting the roles of the respiratory epithelium and the innate immune system in preventing the development of disease. We will also discuss our current understanding of the pathophysiology of aspergillosis in emerging at-risk populations, with a particular focus on patients with cystic fibrosis (CF), chronic obstructive lung disease and severe viral infections.

### Clinical manifestations

The maladies caused by *Aspergillus* spp., collectively referred to as aspergillosis, impose a significant burden on global health [2,39]. One report estimated that *A. fumigatus*-related diseases alone cost the US healthcare system $1.2 billion in 2017 [40]. The development, progression and outcome of infections heavily depend on the condition of the host and its immune system. Rates of *A. fumigatus* infection are associated with levels of exposure; however, specific host factors have also been linked to increased colonisation [41]. Perhaps the most defined of these is prolonged neutropenia, which is heavily associated with the development of invasive aspergillosis and poor patient outcomes despite treatment with antifungals [42,43]. The duration and degree of neutropenia are strongly correlated with the likelihood of developing invasive aspergillosis; in 1984, Gerson [44] estimated that the risk of invasive pulmonary aspergillosis development in neutropenic patients increased by 1% per day for the first three weeks following the onset. Neutrophils are crucial in the defence against fungal pathogens, playing a direct role in killing both conidia and hyphae. Thus, a decrease in their numbers, specifically<500 cells mm^−3^ or a loss of function can have a catastrophic impact on the host [30,45,46]. In addition to defects in immune function, there is also evidence to suggest a genetic basis for *A. fumigatus* colonisation. Indeed, Gago et al. [47] revealed that mutations in the host transcription factor ZNF77 promoted *A. fumigatus* adhesion, germination, and growth via a loss of epithelial integrity.

Currently, there are four distinct types of aspergillosis; invasive pulmonary aspergillosis (IPA), chronic pulmonary aspergillosis (CPA), allergic bronchopulmonary aspergillosis (ABPA) and aspergillus bronchitis (AB) with each affecting different cohorts of patients and inducing varying levels of disease severity [48].

#### Invasive pulmonary aspergillosis (IPA)

IPA is characterised by the dissemination of fungal hyphae in the lung and surrounding tissues [49]. A 2017 study by Bongomin et al. [2] estimated that there were more than 300,000 cases of IPA annually; however, this is likely to be an underestimation as the study also reported that, where data was available, the prevalence of IPA in chronic obstructive pulmonary disease (COPD) patients admitted to hospital ranged from 1.3–3.9%. Recent re-estimations [50] of IPA prevalence in COPD patients have suggested the number of global cases to be between 760,017–2,272,322, which is significantly higher than those reported previously.

IPA is a progressive disease, which poses a serious risk to patient health, with mortality rates typically ranging from 30 to 60% in susceptible populations [51–53]. It is most frequently associated with patients who exhibit a profound defect in their immune function e.g. organ transplant recipients, those receiving chemotherapy and those being treated with corticosteroids [54–57]. Still, there are also emerging populations who do not exhibit immunodeficiency, but who are at increased risk of IPA [58]. These include people with COPD and those who are critically ill in intensive care units (ICU), with this becoming increasingly relevant during the coronavirus (COVID-19) pandemic [59,60].

Typically, IPA begins in the lower respiratory tract where conidia are deposited following inhalation and suboptimal clearance. These conidia then germinate and rapidly produce hyphae, which are capable of invading host lung tissues and disseminating to other organs [32]. Symptoms of the disease are non-specific (shortness of breath, fever, general malaise, coughing) making it difficult to diagnose [61]. This is especially true for patients with non-classic risk factors e.g. those without neutropenia, as there is a lack of clinical suspicion [58].

Radiological imaging is one of the most important tools currently available for the diagnosis of aspergillosis as it is capable of detecting haemorrhagic pulmonary nodules (halo signs), which are characteristic, but not unique to IPA [62,63]. Computed tomography (CT) scans are frequently used to identify early signs of IPA, with the sensitivity of this method reaching>80% in neutropenic patients [64,65]. Advancements in radiological imaging have greatly improved the speed of diagnosis and in turn patient prognosis [66]. However, the diagnostic criteria used in these methods are not specific to IPA and have been observed in other pulmonary disorders including viral infections and angiosarcoma [67,68]. Thus, radiological imaging should be used in conjunction with other methods for a definite diagnosis [69].

Histopathological tissue biopsies demonstrating fungal invasion are the gold standard for diagnosing IPA but can be challenging to obtain, especially from patients who are critically ill [58,70]. Therefore, other tools including PCR and fungal antigen (galactomannan) detection in blood and respiratory samples are also routinely used in the diagnosis of IPA [71,72].

Voriconazole is the primary antifungal used to combat IPA, with treatment recommended to continue for a minimum of 6–12 weeks [36]. Early treatment of IPA is important for positive patient outcomes, therefore it is recommended that patients with strongly suspected IPA should commence antifungal therapy, while a diagnosis is being confirmed [36]. Treatment with voriconazole is generally well tolerated and comparisons to other antifungals, namely amphotericin B, have demonstrated an increased benefit to patient mortality while also causing fewer drug-related adverse effects [73,74]. Unfortunately, certain comorbidities including renal failure can complicate the use of voriconazole, however, the improvements to patient outcomes still justifies its clinical use[75].

#### Chronic pulmonary aspergillosis (CPA)

In contrast to IPA, chronic pulmonary aspergillosis is a relatively uncommon form of aspergillosis [33]. The definition of CPA has evolved in recent years, but it is now characterised by a slow, progressive, cavitating process in the lungs following *A. fumigatus* infection [76]. CPA primarily presents in patients who are not suffering from general immunodeficiency, but who have an underlying lung condition e.g. tuberculosis (TB), COPD or sarcoidosis. Modelling estimates have suggested the global incidence of CPA to be around 17% in newly presenting pulmonary TB cases, making it the most common predisposing risk factor [77]. This is especially true for regions of high TB incidence where it is the underlying cause in~80% of CPA cases [78]. Nevertheless, a lack of quality surveillance data in the literature makes it difficult to estimate the real global burden of CPA [33].

CPA typically presents in an indolent form with many of the early symptoms overlapping with those of predisposing conditions, making diagnosis difficult [79]. Symptoms include coughing, weight loss and fatigue, which are non-specific and indistinguishable from other chronic respiratory illnesses [33,78]. Nonetheless, CPA presents a serious threat to at-risk populations, with the mortality rate of untreated infections reaching 80% after five years [80].

In response, there has been a directed effort towards the generation of specific diagnostic criteria to improve the diagnosis and subsequent treatment of CPA. Denning et al. [33] published detailed clinical guidelines to aid in the detection of CPA, including the identification of at least one lung cavity, which may or may not contain a fungal ball (aspergilloma). They also reported the need for direct evidence of *Aspergillus* infection i.e. microscopy or culture from biopsy, or an immunological response to *Aspergillus* spp. Lastly, symptoms must be present for at least three months and all alternative diagnoses ruled out [33].

Like other fungal diseases, the treatment of CPA with oral azoles largely depends on the patient’s condition. Treatment with itraconazole has been reported to improve symptoms and quality of life in patients with progressive and symptomatic CPA when used for ~4–6 months [81]. Voriconazole has also been used to treat CPA and demonstrates a comparable response rate to itraconazole (61% and 77% respectively) [82,83]. However, Bongomin et al. [84] reported that patients treated with voriconazole typically had a higher burden of disease, both radiologically and clinically, compared to itraconazole treated patients. The appropriate duration for azole therapy in CPA patients remains unknown, however, a retrospective evaluation of treatment outcomes in patients demonstrated that itraconazole and voriconazole were modestly effective for CPA, especially if given for 12 months [84]. Unfortunately, fewer than 50% of patients within the study managed this duration, which further highlights the difficulty in treating CPA [84]. Surgical resection may also be considered for patients with aspergillomas and typically provides a favourable outcome. Though, this treatment is only possible in patients who have good pulmonary function [85]. Intravenous therapies including amphotericin B can also be used as a last resort, following the failure of other treatments or the development of resistance despite their severe side effects [86].

#### Allergic bronchopulmonary aspergillosis (ABPA)

ABPA is caused by a hypersensitivity to *A. fumigatus* allergens and is believed to affect around 4.8 million people worldwide [29]. The repeated inhalation of environmental spores is thought to trigger an allergic IgE-mediated reaction in susceptible individuals [87,88]. ABPA occurs primarily in people with asthma and CF, with the prevalence believed to be around 13% and 9%, respectively [89,90]. Recently, COPD has also been put forward as a risk factor for ABPA, however, the clinical significance of this remains unclear [91,92]. As with other forms of aspergillosis, diagnosing ABPA is difficult due to the overlap of symptoms with other syndromes. Patients often present with non-specific symptoms such as a cough, fever, and general malaise, which are difficult to differentiate [90]. The severity of ABPA varies and acute exacerbations followed by periods of remission are common. If left untreated ABPA can result in chronic fibrotic lung disease [93].

Diagnosis of ABPA relies on a combination of factors including but not limited to: the presence of a predisposing condition i.e. CF or asthma, peripheral blood eosinophilia (≥500 cells/mm3), a positive *Aspergillus* skin test, which reveals immediate cutaneous hypersensitivity to *A. fumigatus* antigens and elevated total serum IgE levels (≥417 IU/mL) [94]. Radiological imaging may also be used to identify central bronchiectasis and mucus plugs in the bronchi [94,95].

The initial steps in treating ABPA involve controlling symptoms of the pre-disposing disease i.e. CF or asthma, followed by treating the *A. fumigatus* infection. Corticosteroids remain the most effective and regularly used treatment for ABPA, with prednisolone being a common first option [96]. A lack of appropriately designed clinical trials means that the exact dosing and duration of treatments are largely undefined, but it is generally accepted that patients should receive therapy for 6–8 weeks [97]. However, the adverse effects of steroid therapy are well documented, with long-term use being associated with immunosuppression and increased susceptibility to infections [98]. Indeed, a study by Fraczek et al. [99] found that corticosteroid treatment was associated with increased fungal burden in patients with asthma.

Second-line therapy with antifungals has also been used, however evidence of its success remains contentious, particularly in patients with CF [100]. A randomised pilot trial by Aaron et al. [101] found no clinical benefit from itraconazole treatment for CF patients whose sputum was chronically colonised with *A. fumigatus*. Alternative azoles such as voriconazole have demonstrated more promise in the treatment of ABPA in people with asthma, with one study reporting a patient response of 70% after three months [102]. Despite this, it remains unclear whether voriconazole will replace corticosteroids as a first-line treatment, due to its associated adverse effects, which have been previously discussed [87].

Omalizumab, a monoclonal antibody against IgE, has also been suggested as a therapeutic alternative, with several studies documenting an improvement in patients [103,104]. Collins et al. [105] reported decreased corticosteroid use alongside a reduction in total serum IgE at twelve months of therapy in patients with ABPA and asthma. Nevertheless, much of the data regarding Omalizumab is preliminary and sample sizes of studies are often small. Thus, further randomised trials are needed to assess the true efficacy of its usage in ABPA [106].

#### Aspergillus bronchitis(AB)

Only recently has AB been re-classified as a distinct form of aspergillosis and thus, there is limited literature regarding its development and prevalence. Common presentations of AB include recurrent chest infections, which are not improved by treatment with antibiotics and significant breathlessness with mucus plugging that is visible with radiological imaging [107]. AB tends to affect individuals who suffer from structural lung diseases, but who are not distinctly immunocompromised [32,107]. Unsurprisingly, it is frequently identified in patients with CF and COPD, causing chronic, non-invasive infections of the lower airway. One cohort study of CF patients suggested the prevalence to be ~ 9%; however, this study contained a relatively small sample size [108].

AB is generally diagnosed following the exclusion of other forms of aspergillosis; a key factor in the diagnosis of AB is that patients do not demonstrate a strong allergic response i.e. elevated serum levels of IgE [109]. Other diagnostic criteria include a symptomatic chronic lower airway disease (>4 weeks), the presence of *Aspergillus* spp. in sputum or BAL and the detection of IgG antibodies to *Aspergillus* spp [107].

As with other forms of aspergillosis, azoles are the primary treatment used in AB. Both itraconazole and voriconazole have been used with success in people without CF, however, there is limited data to support their efficacy in people with CF [107]. Individual case studies have described improvement in CF patients following azole treatment [109,110] but a randomised clinical trial by Aaron et al. [101] reported no benefit to CF patients chronically colonised with *A. fumigatus* following 24 weeks of itraconazole therapy. Thus, the best method for the treatment of AB, especially in CF patients remains unclear and cases should be assessed on an individual basis with a focus on treating the underlying lung disease.

### *Aspergillus fumigatus* virulence determinants

To transition from the saprophytic to the pathogenic form, *A. fumigatus* must adapt to the host environment by deploying a range of virulence strategies. *A. fumigatus* virulence is multifactorial and comprises genes, molecules and properties involved in a range of processes including but not limited to thermotolerance, cell wall composition and maintenance, meeting nutritional requirements, interactions with the host immune system and response to stress (Table 1).

**Table 1.** A selection of *A. fumigatus* genes and molecules involved in virulence. Representative genes from a range of categories including adherence, thermotolerance, nutrition/metabolism, cell wall integrity, interaction with the host immune system and stress response have been highlighted.

PROCESS	GENE/MOLECULE	FUNCTION	REFERENCES
Adherence	aspf2	Laminin binding	[111]
	*cspA*	Extracellular matrix binding	[112]
	*AfCalAp*	Laminin binding	[113]
	GAG	Multi-substrate binding Biofilm formation	[114]
Thermotolerance	*afmnt1*	Mannosyltransferase (cell wall modification)	[115]
	*cgrA*/	Ribosome biogenesis	[116]
Nutrition/metabolism	*alp/aspf13*	Serine protease, degradation of host proteins	[117]
	*creA*	Regulation of carbon catabolite repression	[118]
	*rhbA*	Regulation of nitrogen signalling	[119]
	*cpcA*	Master regulator of amino acid biosynthesis	[120]
	*hcsA, aroC, trpA, ilv3A, ilv3B, hisB, metH, mecA, alaA, lysF*	Amino acid biosynthesis	Reviewed in [121–123]
	*sidA, sidD, sidF, sidH, sidI, sidC*	Siderophore biosynthesis, iron uptake	Reviewed in [124]
	*hapX*	Regulation of iron homoeostasis	[125]
	*zrfC*	Zinc uptake	[126]
	*zafA*	Regulation of zinc homoeostasis	[127]
	*mcsA*	Detoxification of fungal metabolites	[128]
	*pabaA*	Biosynthesis of folate	[129]
Cell wall integrity	*chsG*	Chitin biosynthesis	[130]
	*glfA*	Galactomannan biosynthesis	[131]
	*afmnt1*	Mannosyltransferase (cell wall modification)	[115]
Interaction with the host immune system	*pksP*	Melanin biosynthesis	Reviewed in [132]
	*cat1/cat2/sod1/sod2/sod3*	ROS detoxification	[133,134]
	*gliP*	Gliotoxin biosynthesis	Reviewed in [12]
	*rglT*	Regulation of gliotoxin biosynthesis and self-protection	[135]
	*AfBIR1*	Regulation of programmed cell death	[136]
Stress response	*pkaC1*	Stress and carbon source sensing	[123]
	*pkaR*	Regulation of PKA	[137]
	*mkk2*	Cell wall damage sensing	[138]
	*sskB*	Osmotic stress sensing	[113]
	*ptcB*	Regulation of osmotic stress response	[139]
	*schA*	Regulation of osmotic stress response	[140]
	*cna*	Calcium signalling	[141]
	*crzA*	Calcium signalling	[142]
	*ZipD*	Calcium signalling	[143]
	*srbA*	Oxygen sensor	[144]
	*sskA*	Regulation of osmotic stress	[145]
	*hrmA*	Induction of hypoxic phenotype	[146]
	*HIF-1α*	Immune cell activation	[147]
	*ireA*	Mediator of ER stress via UPR pathway	[148]
	*hacA*	Mediator of ER stress via UPR pathway	[149]
	*pacC*	Alkaline pH response	[150]
Hyphal Growth	*rasB*	Germination and hyphal growth	[151]

#### Adherence

*A. fumigatus* conidia disperse aerially and are inhaled frequently. Thus, the ability of conidia to adhere to host tissues is essential to *A. fumigatus* pathogenicity. Negatively charged carbohydrate motifs and several surface proteins have been implicated in *A. fumigatus* host adherence [152]. Namely, RodA is responsible for the assembly of the rodlet layer that confers hydrophobicity to the conidia and mediates adherence with host collagen and albumin [153,154]. The allergen Asp f 2 and the glycosylphosphatidylinositol (GPI) anchored CspA protein have also been identified as adhesins, with deletion mutants of these proteins exhibiting defective binding to host components *in vitro* [111,112]. Another important conidial-specific adhesin is the extracellular thaumatin domain protein AfCalAp which promotes host laminin binding [113]. In addition to conidia, *A. fumigatus* hyphae are also able to adhere to host cells. Galactosaminogalactan (GAG), an exopolysaccharide that is secreted by *A. fumigatus* hyphae, exhibits multi-substrate adhesive properties [114]. Indeed, Δ*uge3* and Δ*medA* GAG-deficient mutants show reduced adherence to pulmonary epithelial cells *in vitro* [155]. GAG requires deacetylation mediated by Agd3 for fungal adhesion and full virulence [156]. GAG is also an essential component of the extracellular matrix (ECM), making it important for biofilm formation. Subsequently, GAG has also been implicated in antifungal resistance and host immune evasion [114,157].

#### Thermotolerance

*A. fumigatus* is a thermophilic organism that is capable of growing in a wide range of temperatures reaching 50°C [158]. Gene expression profiles at different temperatures have been described in *A. fumigatus* [159,160] but, to date, only two proteins have been found to be necessary for both thermotolerance and virulence. The deletion of α-1,2-mannosyltransferase coded by *afmnt1* causes cell wall damage, which prevents the mutant from growing at 48°C. Attenuation of virulence in a murine infection model was also observed for the mutant [115]. Similarly, the disruption of the *cgrA* gene led to a strain with impaired growth at temperatures above 25°C and showed attenuated virulence in an immunosuppressed mouse model of invasive aspergillosis [116]. CgrA localises into the nucleolus and is involved in ribosome biogenesis, suggesting that new ribosome production is essential for the early phases of *A. fumigatus* infection [161].

#### Nutritional requirements

Like many other pathogens, *A. fumigatus* has developed several strategies to acquire nutrients from its host, with some of these being essential to virulence [162]. One way in which *A. fumigatus* obtains nutrients is by secreting extracellular enzymes that degrade host tissues. These enzymes are speculated to be involved in virulence, however, their redundancy makes them difficult to define [163]. Still, deletion of the *alp*/*aspf13* gene that encodes a secreted serine protease with elastase activity has been linked to mortality reduction in a neutropenic mice model [117]. While carbon is not a limiting factor for *A. fumigatus* infection, CreA, a transcription factor that controls carbon catabolite repression seems to be important for *in vivo* fungal fitness and disease progression [118]. In contrast, nitrogen metabolism appears to have a much more significant role in *A. fumigatus* pathogenicity, with 23% of genes involved in the assimilation and metabolisation of nitrogen required for virulence [164]. For example, the deletion of genes involved in nitrogen metabolism regulation that encode the Ras-related protein RhbA and the CpcA transcription factor causes a reduction in fungal virulence in mice models of invasive aspergillosis [119,120]. One group reported that *cpcA* deletion strains were less virulent in neutropenic mouse models, compared to their wildtype progenitor. The group also demonstrated that reconstitution of the deleted *cpcA* restored wildtype levels of virulence [120].

Amino acids have also been implicated in *A. fumigatus* virulence, with strains lacking genes involved in the biosynthesis of histidine, lysine, tyrosine, tryptophan, isoleucine/valine, methionine, cysteine, and alanine exhibiting impaired virulence in invasive pulmonary aspergillosis animal models [121,165]. Recently, Kerkaert et al. [122] reported that loss of *alaA*, which encodes a protein involved in alanine metabolism, resulted in increased susceptibility of *A. fumigatus* to echinocandin treatment in a neutropenic murine model of IPA. Functional synthesis of other molecules including folate is also necessary for *A. fumigatus* virulence, with the gene responsible for encoding para-aminobenzoic acid (PABA) synthetase, essential for fungal growth in lung tissue of IPA mouse models [129].

In addition to synthesis, protein degradation is also an important aspect of nutrient acquisition within the host. However, degradation of amino acids like valine, methionine and isoleucine can yield toxic metabolites such as propionyl-CoA, which require detoxification. Accumulation of propionyl-CoA, via the loss of 2-methylcitrate synthase (*mcsA*), which catalyses propionyl-CoA degradation impairs fungal growth and limits invasive infections in immunosuppressed mice models [166].

Human pathogens are often limited by the availability of inorganic elements within the host niche. Iron and zinc are the most abundant trace metals in organisms and are essential co-factors for many crucial metabolic processes including *A. fumigatus* growth and virulence [164]. *A. fumigatus* has developed two high-affinity mechanisms for iron intake: i) reductive iron assimilation and ii) siderophore-mediated uptake, with the latter playing a direct role in fungal pathogenesis [124]. Siderophores are iron-chelating molecules that are secreted by the fungus to bind ferric iron before being re-absorbed, providing the fungus with iron [167]. Mutant strains lacking genes involved in siderophore biosynthesis or the transcription factor HapX that regulates iron homoeostasis have been described to have decreased virulence in a murine model of aspergillosis [125]. Similarly, Δ*zrfC* and Δ*zafA* strains that lack genes that encode a zinc transporter and a transcription factor that regulates zinc uptake, respectively, showed reduced virulence in mice models of pulmonary aspergillosis [126,127].

#### Cell wall maintenance

The fungal cell wall represents the first physical barrier of the cell and is in constant interaction with environmental stressors and the host immune system during infection. Consequently, factors involved in the maintenance of cell wall integrity and the dynamic changes in shape and function required for host adaptation could be implicated in fungal pathogenesis [163]. The *A. fumigatus* cell wall is composed of more than 90% polysaccharides with a core skeleton formed by a branched β1,3-glucan linked to chitin, galactomannan, and β1,3- β1,4 glucans. α1,3-glucan and mannan act as a cement while different proteins are bound throughout the structure [168]. As a result, some strains carrying deletions in genes involved in the synthesis of cell wall components have reduced virulence compared to their parental wild-type strains. Namely, deletion of *chsG* (a chitin synthase), *glfA* (involved in the synthesis of galactomannan), *afmnt1* (involved in modification of cell wall proteins and also implicated in thermotolerance) produce strains with reduced virulence properties [115,130,131].

#### Interaction with the host immune system

*A. fumigatus* has a myriad of characteristics that help with the resistance, evasion and weakening of the host immune system. Pigmentation of *A. fumigatus* conidia has been reported to interfere with the host immune system by protecting the cell from reactive oxygen species (ROS) produced by macrophages and neutrophils. In turn, this limits the activation of the complement cascade, interfering with phagosome maturation and masking cell wall components that are involved in recognition by the host immune system [132]. Consequently, deletion of the *pksP* gene (involved in melanin biosynthesis) generates a hypovirulent strain, defective in melanin production in both immunosuppressed and immunocompetent mice and drosophila animal models [169–171].

*A. fumigatus* has also been suggested to interfere with the host immune system via the expression of AfBIR1 [136]. This anti-apoptotic protein has been reported to hinder the activation of fungal programmed cell death (PCD) pathways in neutrophil engulfed conidia. Shlezinger et al. [136] showed that overexpression of AfBIR1 resulted in reduced PCD and enhanced survival under oxidative stress *in vitro*, compared with the parental strain. Moreover, the group also demonstrated that excess AfBIR1 induces a more virulent infection in immunocompetent mice, reflected by higher mortality rates and fungal burden [136]. Lastly, these strains were also associated with a transient increase in total lung leukocytes, neutrophils, and inflammatory monocytes [136].

Catalases and superoxide dismutases also help in the detoxification of ROS produced by host immune cells. The double deletion of *cat1* and *cat2* genes caused reduced virulence in immunosuppressed rats. While the triple deletion of *sod1*, *sod2* and *sod3* genes did not reduce virulence in a murine aspergillosis model, the fungus was more efficiently killed by mice alveolar macrophages [133,134].

*A. fumigatus* is also able to secrete molecules that interfere with the host immune system, the best characterised of which is gliotoxin. Gliotoxin is produced by *A. fumigatus* and its immunosuppressive role relies on the impairment of macrophage phagocytosis, mitogen-activated T cell proliferation, mast cell activation, cytotoxic T-cell response, monocyte apoptosis, and neutrophil function [163]. Deletion of *gliP*, which catalyses the first step in gliotoxin biosynthesis, makes the strain less virulent than the wild-type in non-neutropenic mice models but not in neutropenic models, further emphasising the effect of gliotoxin in neutrophils [12]. Self-protection against gliotoxin is also important *in vivo* as a strain lacking the *rglT* gene, a transcription factor important for regulation of gliotoxin biosynthesis and self-protection, is hypovirulent in a chemotherapeutic murine model of invasive pulmonary aspergillosis [135]. As mentioned before, *A. fumigatus* hyphae secrete GAG during active infection interfering with the host immune system. In particular, it masks the cell wall β-glucan preventing recognition by the host immune cells, induces neutrophil apoptosis and exerts an immunomodulatory effect in host defence cells [114].

#### Stress response

The ability to sense and adapt to the environment is essential for *A. fumigatus* to grow and survive inside the host. To this end, several signalling pathways that shape the cellular response of *A. fumigatus* have been identified and linked to virulence [172].

The cAMP-dependent protein kinase (PKA) is crucial for sensing carbon sources and environmental stress and plays an important role in pathogenesis. Indeed, interruption of either the catalytic subunit PkaC1 or the regulatory subunit PkaR has been observed to reduce fungal virulence in an immunosuppressed mouse model of invasive aspergillosis [123,137]. Due to the essentiality of the fungal cell wall, the ability of *A. fumigatus* to sense and respond to host-specific stressors that can damage that barrier is considered an important virulence trait. In response to cell wall or osmotic stress, cell wall integrity (CWI) and high osmolarity glycerol (HOG) pathways are activated. These signalling pathways consist of the transduction of environmental information and the articulation of cellular response through mitogen-activated protein kinases (MAPKs) [173]. The CWI pathway is composed of three MAPKs (Bck1, Mkk2 and MpkA), which transmit signals by phosphorylating each other sequentially, however, only Mkk2 has been seen to be directly involved in virulence *in vivo* [138]. HOG pathway consists of a two-component system (TSC) and a MAPK cascade (SskB-PbsB-SakA), which ends in SakA (Hog1 homologue) phosphorylation, activating downstream transcription factors, which are articulating the proper response [174]. Although only SskB seems to be required for full virulence, other accessory proteins of the pathway, such as PtcB phosphatase and SchA kinase, which directly regulate SakA phosphorylation, have been implicated in *A. fumigatus* virulence [139,140,175]. For example, Kirkland et al. [145] reported that SskA was an important regulator of the SakA MAPK pathway which drives germination and disease progression.

Calcium homoeostasis is essential for the regulation of several fungal signalling pathways including growth, metabolism, morphogenesis, and cell wall formation [176]. Signalling is mediated by the accumulation and release of calcium from intracellular stores via calcium channels, pumps and transporters [177]. Calcium acts as a secondary messenger modulating the conformation of calcium-binding proteins, such as calmodulin, which activate calmodulin-dependent enzymes including calcineurin (CN) phosphatase [143,178]. The calcium-CN pathway plays a central role in *A. fumigatus* biology, promoting CWI, growth, antifungal resistance and virulence [179]. As a result, the disruption of the genes that encode the CN subunit A, *cnaA*, and the downstream key transcription factor, *crzA*, contributes to a decrease in *A. fumigatus* virulence [141,142]. Several additional transcription factors have also been reported to be important in promoting calcium stress tolerance, but to date only ZipD has been linked to a reduction in virulence in mouse models [143].

Previous work has suggested that the expression of many virulence-related attributes is mediated by the fungal unfolded protein response (UPR), which mitigates endoplasmic reticulum (ER) stress by maintaining an equilibrium between the number of proteins entering the ER and the rate at which they can be folded by the organelle [180]. The accumulation of unfolded proteins in the ER is cytotoxic and has been linked to the activation of several stress response pathways within *A. fumigatus*. This mechanism acts as a regulatory hub for the expression of multiple traits that combine to support fitness in adverse conditions e.g. high temperature, iron starvation and hypoxia within the host [181]. Initiation of the UPR pathway is mediated by the ER transmembrane stress sensor IreA. This molecule facilitates the downstream activation of HacA, which functions as the main transcriptional regulator of the pathway [182]. Loss of both IreA and HacA has been linked to an attenuation of virulence in the host. Feng et al. [148] reported that *ireA* deficient strains were avirulent in an IPA mouse model and showed reduced thermotolerance and increased susceptibility to azole antifungals. Loss of *hacA* also resulted in attenuated virulence, however the impact was not as severe [148]. Further expansion on ER stress and UPR can be found in reviews by Krishnan and Askew [180] and Askew [181].

It is readily accepted that *A. fumigatus* can grow in hypoxic environments, adapting to fit the host niche [183]. Kowalski et al. [146] reported that *A. fumigatus* exhibited a distinct morphology in response to hypoxic conditions, and that this phenotypic alteration was associated with a more virulent infection in murine models of IPA. Further work by this group suggested that this adaptation was mediated by a hypoxia-evolved allele of *hrmA*, which was necessary for the induction of the hypoxic phenotype, and for the increased virulence of infection [146]. Shepardson et al. [11] reported that hypoxic conditions induced changes in *A. fumigatus* cell wall composition, including an increase in β-glucan levels. These findings were later validated *in vivo* using a corticosteroid model of IPA [11]. In addition to altering fungal morphology, there is increasing evidence to suggest that oxygen availability at the site of infection is important in directing the innate immune response [147,184]. Shepardson et al. [11] revealed that hypoxic-grown hyphae induced greater macrophage and neutrophil cytokine and chemokine release; TNF-α and CXCL2, compared to normoxic grown-hyphae. Moreover, hypoxia is frequently associated with an increase in Hypoxia-inducible factor (HIF), which is involved in the activation of immune cells such as neutrophils and macrophages, as well as in the generation of pro-inflammatory cytokines [147]. Ye et al. [185] showed that appropriate expression of HIF-1α was essential for the regulation of Th and Treg cell differentiation via the NLRP3/IL-1β pathway, and in turn, for the effective clearance of *A. fumigatus*. SrbA, a transcription factor that can indirectly sense oxygen levels, is thought to be important for adaptation to the hypoxic host environment [144]. Oxygen sensing has also been implicated in biofilm morphology and promoting antifungal resistance. Analysis of maturing fungal biofilms revealed the formation of spatial gradients of hypoxic microenvironments, which are suggested to drive antifungal resistance. Kowalski et al. [157] reported that mature (12 h) biofilms showed significantly reduced damage when treated at 0.2% O_2_ with voriconazole, amphotericin B, or menadione. Additionally, it was also shown that decreasing oxygen levels towards the base of *A. fumigatus* biofilms increased antifungal drug resistance [157], further highlighting *A. fumigatus* ability to adapt to its environment.

Adaption to the alkaline pH found within the mammalian lung is also crucial for *A. fumigatus* survival and virulence. The alkaline pH-responsive transcription factor PacC has been reported to govern epithelial damage and invasion in leucopenic mice models, with *ΔpacC* ^ATCC^ and *ΔpacC* ^CEA10^ null mutants demonstrating attenuated virulence relative to wildtype strains and reduced fungal-mediated lung epithelial cell damage [150].

## Interactions of *A. fumigatus* with host immune system

**Figure 2.**
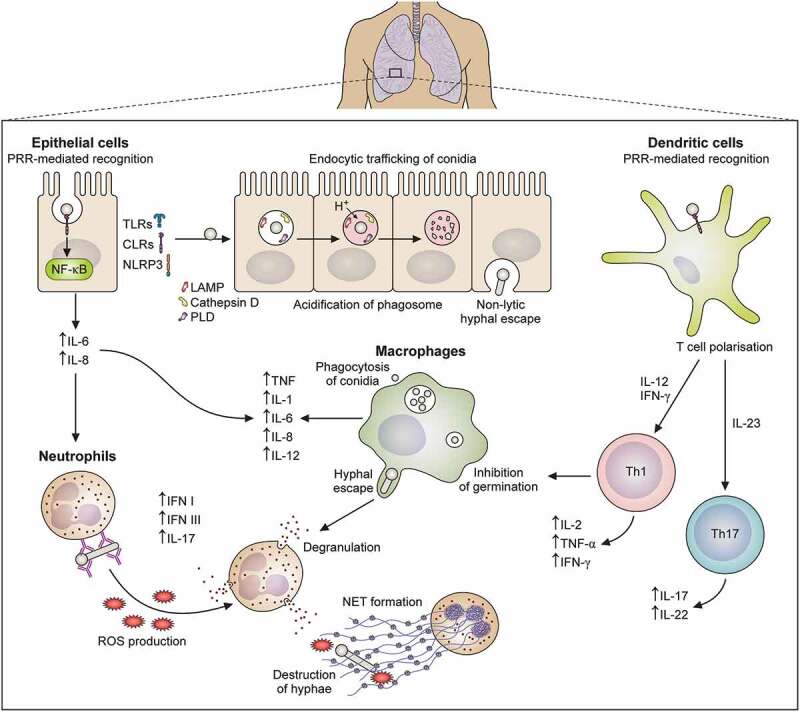
Diagram of innate immune response against *Aspergillus fumigatus*. This figure schematically represents an overview of the information presented in this review regarding epithelial cell, macrophage, neutrophil, and dendritic cell interactions with *A. fumigatus*.

The heterogeneity of *A. fumigatus* infections in at-risk populations suggests that a combination of host and pathogen factors is required for the development of aspergillosis [186–188]. One of the most critical determinants of *A. fumigatus* progression is the innate immune response of the host to inhaled conidia. Here we aim to summarise our current understanding regarding the antifungal potential of the host immune defences soon after spore inhalation, with a specific focus on epithelial cells, granulocytes (neutrophils) and myeloid-derived cells (macrophages and dendritic cells) (Figure 2).

### Interactions of *A. fumigatus* with epithelial cells

Epithelial cells represent the first line of defence against *A. fumigatus* in the lungs. The epithelium is comprised of a variety of lung epithelial cells whose morphology is linked to their functional capabilities [189]. In addition to acting as a physical barrier, these specialised cells are also involved in mucociliary clearance, which prevents conidia from colonising the airway [32]. The secretion of polymeric mucins, primarily MUC5AC and MUC5B, facilitate particle entrapment, while the coordinated beating of cilia on the epithelial cell surface drives the clearance of pathogens [190]. While these mechanisms are normally successful in a competent host, they are much less effective in patients who are immunocompromised or have an underlying lung condition. The inflammation and cilial damage associated with these disorders result in decreased mucociliary clearance, which in turn likely promotes fungal adherence and germination [191].

Adherence to the epithelium is the first step in the development of infection and is mediated by a range of fungal surface components, which differ depending on the morphotype [155]. Our current understanding indicates that swollen *A. fumigatus* conidia bind components of the extracellular matrix of the airway epithelium [113,192,193], initiating spore recognition and internalisation in a time and dose dependant manner.

Spore recognition by lung epithelial cells is primarily mediated via the expression of pattern recognition receptors (PRRs). These include Toll-like receptors (TLR), C-type lectin receptors (CLR) and nucleotide oligomerisation domain-like receptors (NLR) which recognise fungal pathogen-associated molecular patterns (PAMPs) i.e. fungal cell-wall polysaccharides [194,195]. It is the recognition of specific PAMPs by PRRs, which triggers the activation of further antimicrobial mechanisms and initiates fungal clearance.

The exact role of PRRs in the activation of the airway epithelium against *A. fumigatus* is poorly understood. It has been described that *A. fumigatus* induces the expression of Dectin-1, a C-type lectin, in a TLR2-dependent manner, which can mediate the recognition of the fungal polysaccharide β-glucan [150,196,197]. The binding of this molecule to Dectin-1 leads to the activation of two independent signalling pathways, SYK/CARD9 and RAF-1, which induce NF-κB activation and the synthesis of proinflammatory cytokines e.g. Interleukin IL-6, and IL-8 [198,199]. Airway epithelial Dectin-1 has also been implicated in the recognition of both un-opsonised and ficolin-A opsonised *A. fumigatus* conidia, leading to the expression of TNF-α, GM-CSF, IL8, IL-6, IL-17, IL1–3, HBD2, and HBD9 [200]. This has been demonstrated in several *in vitr*o and *ex vivo* systems where the release of these mediators is inhibited by the absence of functional Dectin-1 or TLR2 [201]. Beisswenger, Hess and Bals [202] showed that conidial dsRNA induced inflammatory mediators via TLR3 signalling in human primary bronchial epithelial cells in an internalisation-dependent manner, with these responses believed to be critical for the recruitment of professional phagocytes to the site of infection [203].

In addition to adhesion, epithelial cells are also involved in the uptake of fungal spores, which facilitates intracellular killing [152]. The exact mechanism of this is yet to be elucidated, but it appears to be important in the host antifungal response [204]. To date, it has been suggested that uptake of *A. fumigatus* by airway epithelial cells depends on actin, Dectin-1, cofilin and Phospholipase D (PLD) [205,206]. Internalised *A. fumigatus* spores are then trafficked within phagosomes, which are surrounded by caveolin, flotillin-2, LAMP, RAB5C, RAB8B, RAB7A, 2×FYVE, and FAPP1 [207]. Interestingly, Clark et al. [207] reported that silencing of RAB5C, PIK3C3, and flotillin-2 resulted in a higher population of viable internalised conidia, following 9 hours of challenge with BEAS-2B cells, further highlighting their importance in the endocytic processing of conidia. Destruction of internalised conidia is generally completed one hour after uptake, following fusion and acidification of the spore-containing phagosomes with lysosomes [208–210]. Using high-resolution confocal microscopy, Seidel et al. [211] directly observed that complete fusion and acidification of the phagolysosome prevented the germination of the vast majority of internalised spores. Further work revealed that suboptimal maturation of the phagosome allowed spores to germinate and escape via a non-lytic mechanism [211,212]. Remarkably, observations revealed that during the escape of infected airway cells and penetration of new cells, *A. fumigatus* hyphae were surrounded by the host’s plasma membrane [211]. This mechanism has been suggested to be beneficial to both the host and the pathogen, as it prevents host cells from being lysed and protects the fungus from the host immune response [211].

The inflammatory response against *A. fumigatus* by the lung epithelium is not only critical for the prevention of pathogen growth but also for the activation of downstream immune responses. The formation of the inflammasome and the production of active IL-1β depends on SYK transduction signalling [213]. *Aspergillus* recognition by IL-1 R and TLR also leads to MyD88 signal transduction, which contributes to the expression of NF-KB chemokines by airway epithelial cells [214]. Thus, the coordinated integration of signals triggered by the activation of CLR/CARD9, IL-1R1/MyD88, and TLR/MyD88 pathways are critical for the attraction of phagocytes to the site of infection [215]. Using *in vivo* models of disease, Jhingran et al. [216] demonstrated that protective anti-*Aspergillus* responses depend on the coordinated action of the lung epithelium and haematopoietic cells. While IL1R/MyD88 signalling is initially orchestrated by lung epithelial cells, amplification of the signalling cascade is performed via CARD9 signalling by haematopoietic cells. Additionally, using an *in vitro* model of *A. fumigatus* exposure, Jeong et al. [217] demonstrated that NLRP3 inflammasome-derived IL-1β expression in the airway epithelium may be modulated by the airway epithelial phosphoinositide 3-kinase-δ in a process dependent on the generation of mitochondrial ROS. Looking across all model systems, *A. fumigatus* strains, fungal morphotypes and time points, current published data indicates that spore germination is critical for the induction of robust inflammatory responses by lung epithelial cells in a process leading to the generation of ROS [218]. The interaction of bronchial epithelial cells with germinating but not resting *A. fumigatus* conidia induces IL-8 release, a chemokine critical for the recruitment of neutrophils, via phosphatidylinositol 3-kinase, p38 MAPK, and ERK1/2 activation [219]. Importantly, the migration of neutrophils into the lung airspace is thought to be driven by a hypothetical fungal cell wall protein and does not imply breaching of the epithelial lining [220].

The airway epithelium can also be activated in response to *A. fumigatus* secreted extracellular proteases and toxins which are produced during active hyphal growth on the lung epithelial surface. The secretion of proteases and toxins during fungal growth has been shown to cause serious disruptions in the epithelial barrier and to promote the progression of *A. fumigatus* disease using various *in vivo* and *in vitro* models of disease. Challenge experiments using *A. fumigatus* hyphae show induction in the expression of IL-6, IL-8, IL-1α, IL-1β and TNF-α by bronchial epithelial cells *in vitro* [197]. Exposure of airway epithelial cells to *A. fumigatus* culture filtrates, which are used to recapitulate secreted factors produced during active hyphal growth, have also been shown to induce IL-6 and IL-8. Further work has also shown this effect to be repressed in the presence of serine proteases inhibitors [221,222]. *In vivo* models of exposure to *A. fumigatus* culture filtrates have also confirmed these findings, showing that inflammatory responses in primary airway epithelial cells and murine lungs are dependent on fungal extracellular protease activity [223]. In particular, the fungal proteases Asp f 5 and Asp f 13 have been identified as major contributors in this response, with Asp f 13 depositations being observed in the bronchial submucosa of asthmatic patients [224,225]. Other *in vivo* models have suggested that exposure to Asp f 13 compromises the integrity of the lung epithelium in a process mediated by club cell TRPV4 sensing [226], while Zhang et al. [227] revealed that the *A. fumigatus* toxin, gliotoxin, can dissolve actin fibres leading to the disruption of the lung epithelium.

### Interactions of *A. fumigatus* with neutrophils

Neutrophils are a key component of the primary host response against *A. fumigatus*. In addition to phagocytosing conidia, neutrophils are also heavily involved in preventing the dissemination of fungal hyphae. This is highlighted by the increased susceptibility of neutropenic individuals to invasive aspergillosis [32,191]. Numerous *in vivo* murine models have been established to mimic fungal infections in neutropenic hosts and have shown the severity of neutropenia to positively correlate with fungal burden and mortality following *A. fumigatus* exposure [228].

Exposure of airway epithelial cells to *A. fumigatus* conidia triggers IL-1 R/MyD88 activation, chemokine release and CARD9 activation of neutrophils. This recruits them to the site of infection in a process driven by CXCL1 and CXCL2, which is further enhanced by the production of leukotrienes [216,229–231]. In addition to recruitment, IL-1 R signalling has also been implicated in the maintenance of neutrophil viability, with IL-1 R-deficient mice demonstrating reduced neutrophil survival and higher levels of apoptosis [232]. More recently, it has been demonstrated that extracellular galectin-3, a soluble mammalian β-galactose-binding lectin, is required for neutrophil recruitment and activation in the airways upon *A. fumigatus* infection [233]. Snarr et al. [233] proposed a dual role for galectin-3 in contributing to the anti-*Aspergillus* activity of neutrophils. The study suggested that while neutrophil-intrinsic galectin-3 likely facilitated neutrophil adhesion to the endothelium, extracellular galectin-3 facilitated neutrophil egress to the airways. Another *in vitro* study using epithelial cell/neutrophil co-culture showed that neutrophil recruitment to the airway epithelium required long-term exposure to metabolically active *A. fumigatus* conidia in a process mediated by an unknown cell wall protein [220]. Therefore, it is likely that neutrophil recruitment in response to *A. fumigatus* is fundamentally driven by the damage and subsequent activation of the lung epithelium.

The precise mechanisms by which neutrophils recognise conidia have been well studied with the use of *in vivo* and *ex vivo* models. *A. fumigatus* co-culture with neutrophils isolated from healthy donors or patients with well-defined genetic immunodeficiencies demonstrated that recognition of *A. fumigatus* conidia is mediated by the surface integrin CD11b/CD18, which activates non-oxidative mechanisms of intracellular killing driven by lactoferrin sequestration of iron [234]. Conversely, the extracellular killing of germinated *A. fumigatus* conidia requires recognition of opsonised germlings by antibodies via FC receptors and the production of ROS via NADPH oxidase and myeloperoxidase [234]. Even though Dectin-1 is critical for other immune cells to respond to fungal pathogens, neutrophils do not need to express Dectin-1 for antifungal activity. This suggests that other receptors might be important for triggering CARD9-mediated NF-KB activation [235]. For example, Taylor et al. [236] observed a subpopulation of bone marrow neutrophils that are activated during *Aspergillus* infections in a mechanism regulated by IL-23, IL-6, ROR and Dectin-2. Moreover, using a combination of *in vivo* models of aspergillosis and phenotyping of the anti-*Aspergillus* potential of neutrophils deficient in various TLRs, it has been observed that the activation of different members of the TLR family determines the cellular mechanisms of fungal killing. Namely, TLR2 activation has been shown to facilitate fungal killing by neutrophils via the release of gelatinases and proinflammatory cytokines, while TLR4 mediated killing requires azurophil and myeloperoxidase positive granules [237].

In the lung, neutrophils utilise both intracellular and extracellular mechanisms to directly respond to and eradicate *A. fumigatus* spores and hyphae [238,239]. Intracellular killing of *A. fumigatus* conidia by neutrophils requires the fusion of intracellular granules containing cathepsin, neutrophil elastase and NADPH oxidase activation [240,241]. NADPH activation leads to the production of ROS and the activation of antimicrobial proteases which facilitate *Aspergillus* digestion. It has been described that plasmacytoid dendritic cells can amplify neutrophil effector activity against *A. fumigatus* by regulating NADPH oxidase activity and improving the killing capacity of neutrophils [242]. Moreover, the antifungal potency of neutrophils in response to *A. fumigatus* is also directly enhanced by the production of type I/III interferons by monocytes [243,244].

The extracellular killing of *A. fumigatus* conidia and hyphae requires the production of neutrophil extracellular traps (NETs). NETs are extracellular filamentous structures made of chromatin that are associated with nuclear, cytosolic and granular proteins, which are released in response to infection [245–247]. Results from *in vivo* models indicate that the formation of NETs upon bacterial challenge depends on the type of infectious agent [248]. The capability of neutrophils to produce NETs in response to *A. fumigatus* hyphae has been extensively addressed using *in vivo*, *ex vivo* and *in vitro* models of disease [245,249]. Bruns et al. [245] reported that NET production does not appear to be induced in the presence of *A. fumigatus* conidia. The group proposed that *Aspergillus* may be able to mask components of the conidial cell wall, enabling it to avoid detection [245]. However, this was contradicted by Silva et al. [250] who found that NET release could be induced by *A. fumigatus* conidia in a process dependent on CD11b/CD14 recognition and SYK/SRC/PI3K activation. While there is experimental evidence of their production, the capability of NETs to kill extracellular hyphae remains controversial [247], even though NETs-driven inhibition of fungal growth has been demonstrated *in vitro* [251]. The formation of NETs has been described to depend on the expression of phagocyte oxidase (Phox). Activation of Phox upon PRR recognition of *Aspergillus* β-glucans leads to the production of superoxide anions and downstream oxygen reactive compounds which are critical for fungal killing [252]. Thus, impaired NET formation in patients with chronic granulomatous disease due to impaired Phox function has been linked to the development of aspergillosis [252]. Nonetheless, compensatory mechanisms are thought to exist due to the low prevalence of aspergillosis in this population.

### Interactions of *A. fumigatus* with macrophages

Alveolar macrophages are crucial for the prevention of fungal lung disease, making up ~ 90% of the leukocytes, which are routinely found in the airway [191]. Macrophages are highly plastic, allowing them to tailor their response to the host microenvironment [253]. This results in polarisation towards either proinflammatory, classically activated (M1) macrophages or anti-inflammatory, alternatively activated (M2) [254]. In a healthy host, *A. fumigatus* infection induces polarisation towards M1 macrophages [254]. M1 cells are associated with increased antifungal activity and can readily phagocytose conidia, preventing the development of disease in the patient [32,255]. However, in immunocompromised individuals, macrophages demonstrate a reduced ability to recognise and destroy conidia, which can increase host susceptibility to infection [147].

Alveolar macrophages can sense *A. fumigatus* resting conidia via C-type lectin receptors (DC-SIGN, Dectin-1), pattern recognition receptors such as Pentraxin 3 (Ptx3) and surfactant D [14]. As professional phagocytes, macrophages engulf *A. fumigatus* conidia in an actin-dependent manner [255–258]. Conidia contained within macrophage phagosomes undergo maturation and acquisition of phagolysosomal markers can be detected as early as 30 min after internalisation. Phagosome acidification leads to intracellular degradation of *A. fumigatus* conidia, with complete fungal digestion suggested to require both oxidative and non-oxidative mechanisms [259–261]. However, the importance of oxidative mechanisms in macrophages for fungal killing is under debate with much of the discussion surrounding the role of NADPH oxidase. Grimm et al. [262] reported that NADPH oxidase – deficient mice required >100-fold lower lethal inoculum of *A. fumigatus* versus transgenic mice with monocyte/macrophage-targeted NADPH oxidase activity, suggesting a direct role for NADPH oxidase in fungal killing. Conversely, Cornish et al. [263] suggested that alveolar macrophages do not use NADPH oxidase activity to kill *A. fumigatus* conidia. The group reported that lack of functional NADPH oxidase in gp91^phox−/−^ mice, which were exposed to *A. fumigatus* conidia did not significantly affect the phagocytic ability of the alveolar macrophages or their ability to inhibit the germination of internalised conidia [263]. An extensive review on the role of alveolar macrophages in response to *A. fumigatus* can be found elsewhere (for review [264]).

In recent years, the role of mitochondrial reactive oxygen species (mitoROS) in the antifungal activity of macrophages has been reported [265]. Hatinguais et al. [266] showed that mitoROS production increased in murine macrophages following exposure to *A. fumigatus* conidia and that this process was governed via reverse electron transport (RET). Moreover, the group found that prevention of this process reduced the production of pro-inflammatory cytokines TNF-α and IL-1β in macrophages exposed to swollen conidia of *A. fumigatus* and reduced fungicidal activity [266].

In addition to phagocytosis, macrophages are also important modulators of the immune response [267]. Upon activation, macrophages secrete inflammatory cytokines such as TNF, IL-1, IL-6, IL-8, and IL-12, which are crucial for the recruitment of other immune cells such as neutrophils and dendritic cells, as well as for the initiation and direction of the T helper (Th) immune response [191,268,269]. The induction of inflammation by these cytokines is extremely important for the effective clearance of pathogens, however, dysregulation of this process can cause serious damage to the host [268]. In particular, the dysregulation of TNF production has been implicated in several inflammatory illnesses including sepsis and chronic pulmonary diseases [270]. Thus, it can be said that while the activation of macrophages is essential for the prevention of fungal lung disease, it must be regulated to prevent further damage to the host.

Activation of the macrophage response against *A. fumigatus* via Dectin-1/Syk1 is strongly regulated by the exposure of ß-glucan and melanin on the surface of conidia after removal of the rodlet proteins layer [271–273]. While classical mechanisms and phagosome biogenesis are critical for the intracellular killing of *A. fumigatus* by alveolar macrophages, more recently, it has been suggested that LC3-associated phagocytosis (LAP) is also required for efficient antifungal responses [171,274,275]. To form the LAPosome, SYK alongside TLR2 signalling, contribute to the recruitment of i) the components of the NADPH oxidase complex (NOX2) to the phagosome, generating ROS and ii) ubiquitin-like protein LC3 that plays a critical role in phagosome-lysosome fusion [276,277]. Using both *in vivo* and *in vitro* models of pulmonary aspergillosis, it was shown that LAP is triggered by *A. fumigatus* melanin and that this fungal cell wall protein selectively impairs the recruitment of specific components of the NADPH oxidase to the phagosome, leading to a chronic-granulomatous-like disease phenotype [171,274].

Our understanding of the signalling pathways regulating macrophage response against *A. fumigatus* has evolved dramatically in the last few years, with several *in vivo* and *in vitro* studies shedding light on the mechanistic basis of calcineurin, MyD88, NF-KB, MAVS, NOD and ERK signalling activation [214,218,278–282]. The involvement of these signalling pathways in antifungal immunity has been further highlighted by the increased threat of fungal-associated disease following their modulation. Therapeutics in the form of kinase inhibitors, like those given to cancer patients, have been increasingly reported as a risk factor for the development of pulmonary aspergillosis (reviewed by [283]). The use of IL-6 receptor inhibitors has also been linked with an increased risk of aspergillosis in patients with COVID-19 and will be discussed later in this review [284]. Lastly, a significant number of genetic variants in critical genes for *Aspergillus* sensing and intracellular killing or activation of the downstream immune response have been reported in patients with invasive, allergic, and chronic forms of pulmonary aspergillosis [47,285–290]. However, most of these variants are common in the general population, which suggests that other host factors or pathways might be critical regulators of antifungal mechanisms in macrophages.

### Interactions of *A. fumigatus* with dendritic cells

Dendritic cells (DCs) form a crucial link between innate and adaptive responses and are another key component of antifungal immunity to *A. fumigatus*. There are different subsets of DCs; conventional DCs (cDCs) and plasmacytoid DCs (pDCs) which differentiate from a common DC progenitor, as well as DCs which have been proposed to originate from circulating monocytes (mo-DCs) during inflammation. Each of these subsets has differing functional capabilities and has been implicated in mediating protective inflammation in response to invasive aspergillosis. Here, we discuss the interaction of each of these DC subsets with *A. fumigatus*, and the roles they play in combating invasive fungal disease.

DCs that reside in the lung tissue, predominantly cDCs, are continually sampling the airways which enables them to acquire and process inhaled *A. fumigatus* conidia and to prime fungal-specific T cell responses [291–294].

What triggers DCs to mount inflammatory versus regulatory responses and the downstream mechanisms employed in response to invasive *A. fumigatus* infection is poorly understood. One theory, is that there are distinct subsets of cDCs with differing functional capabilities, referred to as cDC1 and cDC2 [295]. In the context of *A. fumigatus* infection, cDC1s (defined in this study as CD103+) have been proposed to prevent excessive Th17 inflammation, reducing disease severity [296]. This process depends on the production of IL-2 by DCs, as selective knockout of IL-2 in DCs instead results in IL-23 production leading to fatal Th17 inflammation. Conversely, cDC2s, which are dependent on the transcription factor IRF4, are critical in polarising a Th17 CD4+ T cell response (secreting IL-17) to *A. fumigatus* through the production of IL-23 [297]. Indeed Schlitzer et al. [297] showed that prevention of IRF4 transcription resulted in a decreased Th17 response and significantly higher levels of fungal burden. Transfer of *A. fumigatus*-pulsed DCs into naive mice has been shown to confer protective Th1 CD4+ T cell responses secreting interferon (IFN)-γ during *A. fumigatus* infection which decreased fungal burden and enhanced survival [298]. On the other hand, cDCs may also dampen excessive anti-*A. fumigatus* immunity, with one study suggesting that lung intraepithelial DCs inhibit clustering of CD11b+ neutrophils via increased efferocytosis of apoptotic neutrophils which accumulate in the lungs, which would help prevent excessive neutrophilic inflammation and subsequent tissue damage [299]. Ultimately, the discovery of more subsets of cDC1s and cDC2s may give further mechanistic insight into their role in invasive aspergillosis and provide an understanding of how cDCs play opposing roles in promoting Th1 or Th17 inflammation.

In comparison to cDC subsets, pDCs are largely involved in the sensing of intracellular pathogens e.g. viruses and triggering the secretion of type I and III interferons. However, in addition to their role in antiviral immunity, it has also been suggested that pDCs direct antifungal activity [300]. Indeed, large numbers of pDCs are recruited to the lung in mice following intratracheal infection with *A. fumigatus* [301]. Moreover, in otherwise immunocompetent mice, depletion of pDCs results in enhanced mortality following *A. fumigatus* exposure [242,301]. There are several suggested mechanisms by which pDCs may induce protection, including direct fungicidal activity with pDCs spreading over hyphae and preventing their growth [301] and secretion of extracellular traps [300]. A recent study also reported that they can orchestrate other cell types, showing that pDCs are crucial in activating neutrophil NAPDH oxidase to directly kill *A. fumigatus* [242]. Furthermore, using a fluorescent *Aspergillus* reporter (FLARE) conidia, Guo et al. [242] showed that pDC-depleted mice were less effective at killing neutrophil-engulfed *A. fumigatus* conidia, suggesting a fungicidal role for pDCs *in vivo*. Nonetheless, further work is needed to uncover the specific ligands on *A. fumigatus*, which drive pDC activation in the lung.

Finally, inflammatory moDCs have also been shown to be involved in protective anti-*A. fumigatus* immunity. During invasive aspergillosis, large numbers of monocytes are recruited to the lung, potentially forming a moDC population. In a neutropenic mouse model of invasive aspergillosis, moDC numbers were found to rapidly increase over three days post-infection [302]. The chemokine receptors CCR2 and CCR6 play non-redundant roles in recruiting monocytes and allowing expansion of moDCs in the lung. The chemokine receptor CCR2 is expressed on monocytes and moDCs, and depletion of CCR2+ cells in a mouse model of invasive aspergillosis resulted in an increased Th1 but reduced Th17 response, leading to increased mortality [243,303]. Similar depletion models showed that CCR2+ monocytes were associated with impaired production of both type I and III IFNs during fungal infection, as well as decreased early expression of IL-1α, both of which lead to defective neutrophil activation [230,244]. CCR6-deficient mice who have a reduced number of lung moDCs upon infection are also more susceptible to invasive aspergillosis [304]. This shows that both monocytes and particularly moDCs play an important protective role during invasive aspergillosis. Many studies have utilised human moDCs, generated by purifying monocytes from the blood and culturing with GM-CSF and IL-4, to study DC responses against *A. fumigatus*[291–308]. These cells have also been proposed to be more capable of killing *A. fumigatus* conidia in culture compared to undifferentiated monocytes, as well as secreting potent inflammatory cytokines (IL-12p70, IL-23 and IL-27), which promote Th1 responses [293]. Additionally, type 1 IFN (especially IFN-β) can boost moDCs to mediate Th1 responses against *A. fumigatus in* *vitro* [306]. In IPA, moDCs secrete chemokines CXCL9 and CXCL10 [242] factors that can also induce the recruitment of other cell types (e.g. pDCs). Fundamentally, it can be said that monocytes and moDCs are required for fungal killing, priming protective T cell responses and orchestrating immune responses through cytokine and chemokine production. However, disentangling the role of monocytes and moDCs will be difficult due to the ontogenic relationship between the two cell types.

## Mechanistic basis of pulmonary aspergillosis in emerging at-risk populations

### Cystic fibrosis

Cystic fibrosis is a life-limiting genetic disorder caused by a mutation in the cystic fibrosis transmembrane conductance regulator gene (*CFTR*) [309]. CFTR primarily functions as a cyclic adenosine monophosphate (cAMP)-regulated chloride-ion channel, playing an important role in regulating osmolarity at the epithelial surface [310]. CFTR is expressed ubiquitously in epithelial cells, meaning the absence or dysfunction of this protein can induce multi-organ disease [311–313]. Nevertheless, CF is primarily characterised by chronic airway disease; where defective mucus secretion contributes to airway obstruction, inflammation, and a reduction in mucociliary clearance. It is a combination of these factors which ultimately generates a favourable niche for microbial colonisation [314–317]. While bacterial colonisation of the CF airway is well documented [318–320], the burden of fungal pathogens has become increasingly recognised, with *A. fumigatus* being frequently isolated from CF sputum cultures [321,322]. Advancements in next-generation sequencing have greatly improved the identification of fungal DNA in sputum samples, with several studies using this method reporting higher fungal burdens in patients with CF [323,324]. Moreover, whole genome sequencing of *A. fumigatus* strains isolated from patients with CF has revealed the presence of different genotypes, which confer both persistent and transient fungal infections in the CF lung [325]. Screening of *A. fumigatus* strains also uncovered that diploid frequency is high within the CF lung and that genetic variation is created through parasexual recombination [326]. Diploid formation was associated with the accumulation of mutations and haploid offspring which were azole-resistant, leading to the suggestion that parasexual recombination promotes *A. fumigatus* adaptation and persistence in people with CF [326].

Although the exact incidence of *A. fumigatus* infections in people with CF is unknown, previous registry data estimates it to be between 10.3% to 57% [327,328]. Several suggestions have been put forward to explain the increased risk of microbial infections, and in turn, aspergillosis in people with CF. Two of the most cited explanations centre around the composition of the airway surface liquid (ASL), which is a key component of the host defence, regulating antimicrobial activity, ciliary function and mucociliary transport [329]. The “low volume hypothesis” suggests that CTFR-associated defective transport of Cl- and Na+ ions increase water reabsorption, resulting in dehydration of the ASL [330–332]. As a result, this increases mucoviscosity, compressing cilia and impeding mucociliary clearance of inhaled conidia [333]. This hypothesis was supported by Matsui et al. [334], who reported that cultured primary airway epithelia from CF patients demonstrated a depletion of the ASL volume, which resulted in the collapse of cilia and dysfunctional mucociliary clearance. Further work by another group found that rehydration of the ASL was able to restore mucociliary activity, both *in vitro* and *in vivo* [335].

In addition to dehydration of the ALS, a reduction in pH has also been theorised to increase susceptibility to colonisation [336]. CFTR-associated transport of ions such as HCO_3_- and Cl- is important for maintaining mucus composition [337]. The absence of CFTR-dependent HCO_3_- secretion has been implicated in the acidification of CF ASL and the subsequent impediment of antimicrobial activity and innate host defences [338–340]. Smith et al. [341] showed that ASL from healthy individuals readily killed *Pseudomonas aeruginosa*, while ASL isolated from CF patients did not. More recently, Pezzulo et al. [342] reported that ASL pH was reduced in CF pigs and that this was associated with reduced bacterial killing, compared to wild-type pigs. Thus, it could be suggested that the reduction in mucociliary clearance and impairment of host antimicrobial defences associated with CFTR dysfunction could contribute to the establishment of opportunistic pathogens such as *A. fumigatus* in people with CF [310,316,342–344].

In addition to hindering the clearance of conidia, the deposition of thick mucus plaques in the CF airway also contributes to the formation of an anaerobic niche, which can be exploited by adept pathogens, like *A. fumigatus* [345]. Indeed, analysis of persistent *A. fumigatus* isolates from CF patients by Ross et al. [325] revealed increased fitness in low oxygen environments, compared to non-lineage control strains. Pulmonary hypoxia is also commonly observed both in patients with IPA [346] and in *in vivo* models of aspergillosis [347]. Kowalski et al. [146] reported that *A. fumigatus* exhibited a distinct morphology in response to hypoxic conditions and that this phenotypic alteration was associated with a more virulent infection in murine models of IPA. Similarly [348], showed that the outcome of IPA in a murine model could be improved via a reduction in hypoxia.

Effective recognition of pathogens plays a crucial role in the initiation of the host immune response [349,350]. Ptx3 is a pattern recognition receptor, which is expressed in a range of cells including alveolar epithelial cells, monocytes and macrophages and has been shown to bind to *A. fumigatus* conidia, as well as to other common lung pathogens like *P. aeruginosa* and *Staphylococcus aureus* [351,352]. Ptx3 levels were found to be lower in sputum taken from CF patients, suggesting that there may be aberrations in pathogen recognition in CF individuals. This was supported by Garlanda et al. [353], who revealed that *Ptx3*-deficient mice showed defective recognition of *A. fumigatus* conidia by alveolar macrophages. Therefore, defective recognition of pathogens could be another factor, which predisposes CF individuals to fungal colonisation.

Internalisation and intracellular killing of inhaled pathogens has also been recognised as a potent antifungal mechanism in the host airway as previously described [354,355]. Several studies suggest that dysfunction of these mechanisms could pose a risk for *A. fumigatus* colonisation, reporting defective internalisation of *A. fumigatus* conidia in both phagocytes and epithelial cells of CF patients. Chaudhary et al. [356] demonstrated that IB3 cells; an epithelial cell line with a CFTR mutation, showed reduced binding and uptake of *A. fumigatus* conidia compared to corrected wild-type cells. The group also reported that the CF cells were less effective at killing internalised conidia [356]. The exact mechanism of fungal internalisation and killing is yet to be elucidated, however, Seidel et al. [211] demonstrated that most internalised conidia are killed following fusion to the acidic lysosome. Interestingly, one study by Di et al. [357] reported that a lack of CFTR is associated with aberrant acidification of the lysosome in alveolar macrophages and in turn, the poor phagolysosomal killing of pathogens. However, this theory was directly opposed by Law et al. [358] who reported that there was no significant difference in phagolysosomal pH between CF and healthy macrophages. Despite the lack of consensus surrounding the exact mechanism, it can be said that CF patients do exhibit reduced internalisation and intracellular killing of *A. fumigatus*, which may contribute to their increased risk of aspergillosis.

Conidial adhesion takes place via the binding of specific sugars or lectins on the conidial surface to host cells and components of the ECM [359]. The ECM is largely composed of fibrous proteins such as collagen, which has been directly shown to facilitate the adhesion of *A. fumigatus* conidia [360,361]. Collagen levels are reported to be significantly higher in the alveolar tissues of CF patients, with the characteristic loss of epithelial integrity and subsequent exposure of the basement membrane in CF patients increasing its availability [362,363]. Thus, it could be proposed that the increased accessibility of collagen in CF patients promotes fungal colonisation by facilitating the more effective adhesion of conidia [364].

Lastly, the effective clearance of pathogens like *A. fumigatus* also relies on a balance between Th cell responses [365]. Appropriate activation of the Th1 pathway is required for the recruitment of neutrophils and for the initiation of phagocytosis, which is a crucial component of the antifungal defence [366]. However, CF patients exhibit a distinct favouring of a Th2 antifungal response, showing increased production of IL-4, IL-13, and IgE [367]. Previous studies have shown that CFTR−/− mice produce significantly higher levels of IgE compared to their normal counterparts when exposed to *A. fumigatus* antigens [368]. Further work suggested that the increased IgE levels were due to a polarisation towards a Th2 response, driven by a dysfunctional CFTR channel in T cells [368]. Similar studies also found that there was a substantial difference in the secretion of Th2 cytokines such as IL 4 and IL-13 from CTFR deficient CD4+ and CD11b+ cells [369]. Thus, it appears that CFTR dysfunction may play a part in disrupting the balance between the Th1/Th2 response, which likely contributes to the establishment and persistence of *A. fumigatus* in CF patients.

### Chronic obstructive pulmonary disease (COPD)

COPD is a progressive lung disease, and one of the leading causes of morbidity and mortality globally [370,371]. The burden of COPD on public health is such that in 1997, the Global Initiative for Chronic Obstructive Lung Disease (GOLD) was formed, aiming to bring attention and management to the disease [372]. Clinically, COPD can be characterised by chronic inflammation, the obstruction of airflow and structural abnormalities in the lungs and airways [373,374]. People with COPD are often prone to exacerbations, which are heavily associated with microbial colonisation and a decline in functional status [375–377]. Hammond et al. [50] highlighted the burden of aspergillosis in COPD, revealing that the global prevalence of COPD (GOLD grade II-IV) was 7.39% and that 13.6% of this population demonstrated sensitisation to *Aspergillus*.

The mechanistic basis for the increased susceptibility of people with COPD to *A. fumigatus* infection remains largely unknown. The most prominent clinical feature of COPD is chronic inflammation, which arises from the hyper-reaction of both the innate and adaptive immune systems. This overreaction is marked by an influx of alveolar macrophages, neutrophils, and T cells [378].

Disease phenotypes in COPD vary widely between patients, yet despite this heterogenicity, all patients exhibit airway neutrophilia [379,380]. Neutrophils are an important marker of systemic inflammation and may be used as an indicator of disease severity and progression in COPD [381]. Analysis of blood neutrophil counts from a large population-based COPD registry by Lonergan et al. [382] found that high neutrophil counts were associated with an increased frequency of exacerbations and mortality in people with COPD. Another study by Bafadhel et al. [383] identified a positive correlation between *A. fumigatus* colonisation and neutrophils in patient sputum, providing further evidence for their role in fungal disease. As previously discussed, a lack of neutrophils (neutropenia) is a significant risk factor for invasive aspergillosis [191]. Their importance has been highlighted in both patients and mice models; a case study of patients with acute leukaemia revealed that profound neutropenia of more than three weeks increased the risk of invasive aspergillosis four-fold [44], while a mice model infected with *A. fumigatus* conidia, showed that depletion of neutrophils was associated with high mortality rates and hyphae-induced lesions in the lung [384]. Yet, despite their importance in the antifungal response, excessive neutrophil recruitment often contributes to airway destruction via the release of proteases like neutrophil elastase and the promotion of a pro-inflammatory environment [385].

Frequent activation of the inflammasome, immune dysregulation and chronic colonisation by pathogens appear to drive a persistent auto-inflammatory response in people with COPD, with a cohort study revealing a higher sensitisation in COPD patients to a range of allergens, including fungal, compared to healthy populations [92,386]. Nonetheless, it remains to be seen whether sensitisation to *A. fumigatus* is a consequence of an already damaged airway, or if it is promoted via persistent *A. fumigatus* colonisation [383].

Effective clearance of pathogens like *A. fumigatus* also relies on a balance between different subsets of Th cell responses [365]. The role of Th1 cells in antifungal immunity is well described, in both human and murine models [387,388]. Th1 cells are responsible for the secretion of important immune factors like IFN-γ, GM-CSF, and TNF, which are required for phagocyte maturation and killing. Specifically, IFN-γ has been shown to arrest fungal growth by triggering the production of ROS in macrophages *in vitro* [389]. Th17 cells have also been implicated in antifungal immunity, playing an important role in the activation and recruitment of neutrophils to the site of a fungal infection via the production of IL-17 [390]. A study investigating *A. fumigatus* infection in immunocompetent rats revealed that higher IL-17 levels were associated with faster resolution of infection [391]. Involvement of the Th17 pathway is also becoming increasingly recognised in the context of allergic lung disease and dysregulation in this pathway has been linked to increased disease severity in in people with COPD [392,393]. Characterisation of the Th cell cytokine profiles from sputum samples of 87 COPD patients during acute exacerbations revealed that a bias towards Th1 response and away from Th17 was associated with poor patient prognosis including increased duration of hospital stays and frequency of exacerbations. Conversely, patients who had adequate levels of both Th1 and Th17 exhibited better clinical outcomes [394]. The authors hypothesised that insufficient levels of IL-17 increase susceptibility to infections and in turn acute exacerbations. Interestingly, Lindén et al. [395] also reported that lower IL-17 levels were related to pathogen colonisation in COPD patients. Thus, the increased incidence of aspergillosis in COPD patients, could in part be due to their impaired ability to produce an appropriate Th17 response.

As discussed, successful recognition, uptake and phagolysosomal killing of conidia by alveolar macrophages and neutrophils is crucial in preventing the establishment of *A. fumigatus* [377]. Recent work by Bertuzzi et al. [355] showed that these processes are aberrant in people with COPD. *In vitro* analysis of primary human airway epithelial cells derived from COPD donors demonstrated an increased ability to internalise *A. fumigatus* conidia, compared to healthy counterparts. Using imaging flow cytometry and live-cell microfluidic imaging, the group also observed that COPD airway epithelial cells exhibited a decreased ability to kill conidia intracellularly [355]. Together these results suggest that defective clearance and killing of *A. fumigatus* conidia is a serious risk factor for aspergillosis in COPD.

Like CF, damage to the epithelial barrier and defects in mucociliary clearance in COPD patients increase the risk of *A. fumigatus* colonisation [396]. As previously discussed, maintaining a compact epithelial barrier is crucial to preventing the transepithelial crossing of inhaled pathogens and irritants [397]. Loss of barrier function is a common observation in COPD, with disruption of epithelial junctions causing gaps in the tissue. Indeed, Heijink et al. [398] showed that bronchial epithelial cells from patients with COPD had a reduced capacity to form tight junctions when cultured in air–liquid interface *in vitro*. Using immunofluorescence, the group investigated the junctional localisation of two tight junction proteins, occludin and ZO-1 and, found that the expression of both was decreased in the COPD cells [398,399].

The excessive inflammation associated with COPD also results in airway remodelling, which can impose further deleterious structural changes to the epithelium [400]. These changes include subepithelial fibrosis following a rise in ECM deposition and an increase in bronchiolar smooth muscle, which has been known to enhance airway hyperreactivity to irritants [381,401]. Goblet cell hyperplasia has also been observed, resulting in excessive mucus production [381]. Indeed, the hypersecretion of mucus is a common characteristic of the COPD airway, and one which is believed to promote microbial colonisation [402,403]. It is readily accepted that excessive levels of mucus can impede mucociliary clearance and inhibit the effective clearance of inhaled pathogens, promoting fungal colonisation [404]. Even though fungal proteases are capable of inducing mucin gene expression, specifically MUC5AC and MUC5B, Wu et al. [405], measured MUC5AC and MUC5B transcripts in epithelial cells following co-incubation with different concentrations of *A. fumigatus* extracts for 6 and 24 hrs, reporting that increase in MUC5AC was both time and dose dependant. This could suggest that there is a continuous feedback loop, in which the hypersecretion of mucus promotes *A. fumigatus* colonisation, facilitating the secretion of fungal proteases and further driving mucus production and ultimately, disease [401].

Another predisposing risk factor for aspergillosis is prolonged treatment with broad-spectrum antibiotics and corticosteroids [27,406,407]. Ng, Robson and Denning [408] found that the growth rate of *A. fumigatus* increased 30–40% following exposure to pharmacological doses of hydrocortisone. More recently, it has been shown that corticosteroid use can alter the lung microbiome, decreasing microbial diversity [99]. This dysbiosis is associated with the enrichment of specific microbial populations, including *Haemophilus spp*. and *Staphylococcus spp* [409]. Thus, it could be that corticosteroid-mediated disruptions to the microbiome in patients with COPD provide a niche for opportunistic pathogens such as *A. fumigatus* to proliferate.

### Viral-associated pulmonary aspergillosis

Our knowledge of the pathophysiology of pulmonary aspergillosis has changed drastically in recent years and, it is now appreciated that individuals suffering from severe viral respiratory infections such as those caused by Influenza virus [410], cytomegalovirus [411] or more recently SARS-CoV2 [412] have an increased risk for the development of aspergillosis. Indeed, during the H1N1 pandemic aspergillosis was a complication in 14–32% of critically ill patients [410,413] and the mortality risk of influenza-associated pulmonary aspergillosis (IAPA) was double that of patients without coinfection. During the ongoing coronavirus pandemic a similar picture has been reported. In a multinational study carried out by the European Confederation of Medical Mycology (ECMM), the overall prevalence of COVID-associated pulmonary aspergillosis (CAPA) was 10.7% and decreased survival was observed in patients with CAPA versus those with only COVID-19 [414]. More recently, a comprehensive analysis of vital statistical data from the US has demonstrated a significant increase in fungal-related deaths between 2020 and 2021, with most of them attributed to COVID-19-associated fungal disease [415]. This data highlights the need to better characterise the epidemiology of viral-associated fungal diseases and guide public health interventions to minimise the associated morbidity and mortality [416].

Most of what we know about fungal disease in patients with severe viral infections has been based on studies focused on understanding the pathophysiology of secondary bacterial infections in patients with viral disease. Superinfections caused by *Streptococcus pneumonia* and *Staphylococcus aureus* have long been recognised as complications of severe influenza and similar figures have been reported during the COVID-19 pandemic [417]. In these patients, viral-mediated epithelial cell damage and dysregulation of the immune defences at the site of viral infection have been suggested to increase susceptibility to secondary fungal disease [418]. In a recent publication, using murine models of *Aspergillus*-Influenza co-infection, it was reported that there is a decrease in neutrophil recruitment to the lungs, which may underpin increased fungal and viral burdens [419]. Using a similar model of combined infection, it was reported that timely oseltamivir treatment prevented severe influenza pneumonia, thus reducing the probability of mortality due to pulmonary aspergillosis [420]. Similarly, neuraminidase inhibitors, which are used to treat influenza virus infections, may decrease the capacity of PBMCs to kill *A. fumigatus* [421].

As discussed above, the functionality of the airway epithelium is critical in preventing the establishment of pulmonary infections. Viral replication in the airway epithelium can cause damage to airway epithelial cells and surrounding tissues, leading to the loss of epithelial integrity and impediment of mucociliary clearance. In turn, this generates a niche for subsequent infections by inhaled pathogens [417]. However, aspergillosis in patients with severe viral infections occurs more rapidly than in other patients with damaged airways who are susceptible to aspergillosis e.g. asthma or COPD. Therefore, it appears that alternative mechanisms may be involved in the development of aspergillosis.

Viral-associated pulmonary aspergillosis is often diagnosed in the first days after ICU admission and in some cases, on day 0 [414,422]. This suggests that patients might be colonised before hospital admission, rather than developing the infection after admission [423]. We have previously reported that fungal colonisation is necessary for the development of pulmonary aspergillosis [424] and that *Aspergillus* colonisation might be genetically regulated [47]. Airway epithelial cells can bind, internalise and kill *A. fumigatus* spores. While those responses might be protective, it has been reported that in patients susceptible to aspergillosis, those mechanisms are aberrant [47,355,356]. To understand whether viral infection regulates airway epithelial cell responses against fungi, we developed an *in vitro* model of fungal-viral coinfection of AECs that has allowed us to determine a synergistic inhibition of antiviral and antifungal airway epithelial cell response. Subsequently, this leads to fungal survival and increased viral replication [425] as previously reported in *A. fumigatus*-cytomegalovirus coinfection of dendritic cells [426] and SARS-CoV2 – *A. niger* coinfection of the lung epithelium [427].

Many respiratory viruses have evolved ways to evade recognition and prevent immune signalling [428]. Influenza has been seen to inhibit type I IFN signalling pathways, which are a crucial component of the anti-viral response [429]. IFNs are also involved in the defence against fungal pathogens [430,431]. Espinosa et al. [244] reported that type I and III IFNs were critical for the activation of host neutrophils in response to *A. fumigatus*. The study also revealed that IFNs were necessary for the generation of ROS in neutrophils, which are a primary mechanism of fungal killing [244]. Therefore, fungal-mediated inhibition of IFN responses might promote copathogenesis [425].

Infection by respiratory viruses has also been reported to increase mucus secretion in the airways using *in vitro* and *in vivo* models of infection [432,433]. Murine models of *S. pneumoniae* and Influenza virus infection have reported that viral-induced reduction of tracheal mucociliary velocity limits bacterial clearance, thus facilitating bacterial colonisation and the development of disease [434]. Another factor that might be facilitating colonisation of the airways is viral-mediated changes to the ECM, exposing cell receptors and basement membrane components to which microbes can adhere [435]. Indeed, this is the case for SARS-CoV-2, where infection has been shown to alter the expression of ECM components like collagen, fibronectin, and laminin, which could promote *A. fumigatus* conidial adhesion [204,436].

The use of mechanical ventilation and corticosteroids in the treatment of severe respiratory viruses has also been cited as a risk factor for the development of aspergillosis [437]. Rouzé et al. [438] reported a 4.1% and 10.2% incidence of invasive pulmonary for patients who were mechanically ventilated following COVID-19 and Influenza infection, respectively. Still, the true incidence is likely to be much higher, given the difficulty of diagnosing *Aspergillus* infections and the difficulties to perform bronchoalveolar lavage in COVID-19 patients [439,440]. Another observational study of ICU patients also revealed that high-dose corticosteroid treatment and ventilation were associated with *A. fumigatus* colonisation and a higher mortality rate [406]. Corticosteroids are regularly used to treat viral-induced inflammation in critically ill patients due to their anti-inflammatory and immunosuppressive capabilities [441,442]. However, as a consequence, patients may endure an increased susceptibility to secondary infections. A retrospective study by Wauters et al. [413] reported corticosteroid treatment as an independent risk factor for the development of invasive pulmonary aspergillosis superinfections in ICU patients with Influenza. On the contrary, a prospective study by Marta et al. [443] reported no relationship between the appearance of CAPA, mechanical ventilation and corticosteroid treatment. However, the authors acknowledged that dexamethasone was administered to all patients sampled in this study as part of their treatment plan, which could have impacted the results [443,444]. Another study, which used an *ex vivo* whole blood challenge assay with *A. fumigatus* antigens showed that patients with COVID-19 had deficient anti-mould responses in a process that might not depend on corticosteroid treatment [445]. Thus, the extent to which corticosteroid therapy and invasive mechanical ventilation contribute to the increased incidence of aspergillosis in patients infected with respiratory viruses remains unclear. The pathogenesis of respiratory viral and fungal coinfections has been reviewed in [446].

Fungal coinfections occur in a scenario of limited therapeutic options and clinical studies like the ones discussed above, suggest that some treatments might tip the balance from one disease to the other. Triple RNA-seq analysis of dendritic cells coinfected with cytomegalovirus and *A. fumigatus* indicates a differential transcriptional regulation of certain *A. fumigatus* genes in coinfection compared with a single infection [426]. This suggests that *A. fumigatus* might deploy different adaptation mechanisms to cause disease in patients with severe respiratory viral infections. Mortality rates in patients with pulmonary aspergillosis following viral disease are disproportionately high and there are many outstanding questions concerning the pathophysiology of this. A recent publication suggests that severe viral infections caused by Influenza virus and SARS-COV2 affect transcriptional responses regulating epithelial cell integrity, the capacity to kill phagocytosed *Aspergillus* spores and the mechanisms of neutrophil-mediated hyphal destruction [447]. However, deducing the specific fungal factors, which are contributing to the pathogenesis of viral-associated pulmonary aspergillosis is critical to developing targeted prevention therapy.
